# Dynamic changes in cell-surface expression of mannose in the oral epithelium during the development of graft-versus-host disease of the oral mucosa in rats

**DOI:** 10.1186/1472-6831-14-5

**Published:** 2014-01-16

**Authors:** Hironori Hanada, Jun Ohno, Kei Seno, Nobutaka Ota, Kunihisa Taniguchi

**Affiliations:** 1 Department of Morphological Biology, Division of Pathology, Fukuoka Dental College, Tamura, Fukuoka 2-15-1, Japan; 2 Department of General Dentistry, Division of General Dentistry, Fukuoka Dental College, Tamura Fukuoka, 2-15-1, Japan; 3 Department of Oral and Maxillofacial Surgery, Division of Oral Oncology, Fukuoka Dental College, Tamura, Fukuoka 2-15-1, Japan

**Keywords:** Graft versus host disease, *Lens culinaris* lectin, Mannose-binding protein, CD8^+^ lymphocytes

## Abstract

**Background:**

The role of cell-surface glycoconjugates in oral mucosal graft-versus-host disease (GVHD) is still unclear, even though molecular changes in the oral epithelium are essential for the pathogenesis of these lesions. In this study, we investigated changes in the binding of mannose (Man)-specific *Lens culinaris* lectin (LCA) in the oral mucosa of rats with GVHD.

**Methods:**

Lewis rat spleen cells were injected into (Lewis x Brown Norway) F_1_ rats to induce systemic GVHD, including oral mucosal lesions. Tongue and spleen samples were evaluated using lectin histochemistry, immunohistochemistry, Western blotting, transwell migration assays and Stamper-Woodruff binding assays.

**Results:**

Binding of Man-specific LCA expanded to the epithelial layers of the tongue in GVHD-rats. An expansion of LCA binding was related to the increased expression of mannosyltransferase in the oral mucosa. CD8^+^ cells, effector cells of oral mucosal GVHD, expressed mannose-binding protein (MBP) and migrated to the medium containing Man in the transwell migration assay. Adherence of CD8^+^ cells to the oral epithelium could be inhibited by pretreating CD8^+^ cells with MBP antibody and/or by pretreating sections with Man-specific LCA.

**Conclusions:**

Increased expression of Man on keratinocytes leads to the migration and/or adhesion of CD8^+^ cells in the surface epithelium, which is mediated in part by the MBP/Man-binding pathway during the development of oral mucosal GVHD.

## Background

Graft-versus-host disease (GVHD) is a complication that can occur after a hematopoietic stem cell or bone marrow transplant, which the newly transplanted donor cells attack the transplant recipient’s body. GVHD is characterized by selective epithelial inflammation which affects the mucocutaneous organs, gastrointestinal tract, and liver [1]. Both clinical and experimental studies have identified oral mucosa as one of the critical sites affected by GVDH [2,3]. Histopathological changes in mucocutaneous GVHD include satellitosis, in which lymphocytes form clusters around dyskeratotic and/or necrotic keratinocytes (KCs). Lymphocytes, particularly CD8^+^ cells, migrate from the perivascular interstitium into the overlying epithelial layer and induce degenerative changes in KCs, suggesting that determining the nature of KC destruction may aid in understanding the pathogenesis of mucocutaneous GVHD [4]. During this process, expression of specific molecules, such as MHC class II and intercellular adhesion molecule-1 (ICAM-1), occurs in epithelial KCs to interact with effector cells [5-8]. Particularly, increased expression of ICAM-1 on KCs leads to the migration of effector cells into the surface epithelium, which is mediated in part by the ICAM-1/ lymphocyte function-associated antigen-1 (LFA-1) pathway [3].

Recent progress in glycobiology has revealed that cell surface glycoconjugates play an essential role in recognition events. Lectin probes have typically been used to detect cell surface glycoconjugates, because lectins are defined as carbohydrate binding proteins other than enzymes or antibodies (Abs) [9]. They are involved in diverse biological processes in many species, such as clearance of glycoproteins from the circulatory system, adhesion of infectious agents to host cells, recruitment of leukocytes to inflammatory sites, and cell interactions in the immune system in conjunction with malignancies and metastasis [9]. Lectin binding is modified during the process of epithelial differentiation and injury stimulation of the skin and oral mucosa [10-13]. Among those lectins, *Lens culinaris* lectin (LCA), known as a mannose (Man) binder, possesses unique binding specificities, with a biantennary core of 1,6-fucosylated oligosaccharides and glycans of human serum transferrin and α-fetoprotein [14]. Cell surface Man is also a ligand for mannose-binding protein (MBP), which functions in opsonization of microorganisms for phagocytes and cell-mediated cytotoxicity-antitumor activity [14,15]. These studies have led to the hypothesis that determining changes in Man expression on KCs will aid in understanding the pathogenesis of oral mucosal GVHD. Our approach to this premise has been to focus on cell-surface Man expression by KCs and its interaction with MBP in rat oral mucosal GVHD of the rats. Our study had three goals: (1) to determine cell-surface Man expression by KCs using Man-specific LCA, (2) to investigate whether CD8^+^ cells, effector cells in oral mucosa GVHD, migrate to a Man-containing medium, and (3) to determine whether Man expression by KCs mediates binding of MBP-expressing CD8^+^ cells to KCs. The results indicate that during the development of oral mucosal GVHD in rats, increased expression of Man by KCs leads to the migration and/or adhesion of CD8^+^ cells in the surface epithelium, mediated in part by the MBP/Man-binding pathway.

## Methods

### Rats

Adult female inbred Lewis (LEW, RT1^l^) and Lewis × Brown Norway F_1_ hybrid (LBNF_1_, RTl^1/n^) rats weighing 250 to 350 grams were purchased from Kyudo Co. (Saga, Japan). The animal studies were conducted in accordance with protocols approved by the Animal Care and Use Committee of Fukuoka Dental College.

### Induction of GVHD

Spleens removed from LEW rats were dissected in Hanks’ solution, forced through a stainless steel sieve, and filtered through a nylon mesh (Cell Strainers; BD Biosciences, CA, USA). The cells were washed three times in Hanks’ solution and resuspended at 10^8^/ml in RPMI-1640 medium with 10% fetal calf serum. Cell viability was determined by trypan blue exclusion analysis. GVHD was induced by a 3-ml intraperitoneal injection of 3 × 10^8^ cells into LBNF_1_ rats. Untreated LBNF_1_ rats and LBNF_1_ rats injected with an equal number of syngeneic LBNF_1_ splenocytes were used as controls. All rats were weighed daily and carefully observed for clinical signs of disease.

### Assessment of GVHD

Clinical assessment of GVHD was determined by weight loss and the development of cutaneous or mucosal erythema, especially on the ears, nasal mucosa, foot-pads, and lips. Both clinical conditions appeared on day 10 after injection and became severe thereafter. A spleen weight assay was performed at autopsy to confirm the immunological assessment of GVHD. After day 10, the experimental rats showed remarkable splenomegaly as an overexpression of GVHD-related immune response [16]. All control animals survived and appeared healthy.

### Tissue preparation

The whole tongue was excised 1–21 days after injection from five animals in each treatment group. Half of the tongue specimens were fixed in 4% paraformaldehyde in phosphate-buffered saline (PBS). Paraffin sections (4 μm) were then stained with hematoxylin and eosin (H&E) to examine histopathological changes. The other half was immediately frozen in liquid nitrogen, and serial frozen sections were used for immunostaining, lectin histochemistry, and *in vitro* adhesion assays.

### Lectin histochemistry

LCA, *Ulex europaeus* I (UEA-1), and *Archis hypogaea* (peanut: PNA) were used for detection of α-D-Man, α-L-fucose, and galactose β1,3galactosamine, respectively (Vector Laboratories, Inc., Burlingame, CA, USA). LCA binding was detected using the Alexa Fluor 568 - labeled streptavidin biotin method. Acetone-fixed frozen sections were incubated with biotinylated LCA (25 μg/ml; Vector Laboratories) for 30 min and Alexa Fluor 568-labeled streptavidin (1:400; Molecular Probes, Eugene, OR, USA) for 30 min at room temperature. Histochemical controls substituted unconjugated LCA for the LCA-biotin conjugate and reaction with biotinylated LCA inactivated by its specific sugar inhibitor (D-mannose, 2 mM; EY Laboratories, Inc., San Mateo, CA, USA).

### Immunohistochemistry

Abs used in this study were as follows: (a) a rabbit polyclonal Ab against ALG11, reactive with mannosyltransferase (1:300; Novus Biologicals, Littleton, CO, USA), (b) a rabbit polyclonal Ab against LMAN2, reactive with MBP 2 (1:100; Aviva Systems Biology, San Diego, CA, USA), (c) a mouse monoclonal Ab against 1A29, reactive with ICAM-1 (1:100; Cederlane Lab., Ontario, Canada), and (d) a mouse monoclonal Ab against OX-8, reactive with CD8 (1:100; Cederlane Lab.). Acetone-fixed frozen sections were first incubated with normal rabbit serum to decrease nonspecific binding and then reacted with one of the Abs, overnight at 4°C. Sections were then incubated with alkaline phosphatase-conjugated anti-rabbit Ab or anti-mouse Ab (1:150 dilution; DakoCytomation, Tokyo, Japan) for 45 min at room temperature. Immunohistochemical reactions were visualized using 5-bromo-4-chloro-3-indolyl phosphate/ nitro blue tetrazolium chloride solution (BCIP/NBT solution; DakoCytomation). As a control, sections were treated with normal rabbit IgG instead of the first set of Abs.

### Western blotting

Total protein was extracted from spleen and tongue specimens in control or GVHD rats using ice-cold cell lysis buffer (20 m M Tris–HCl, pH 7.5; 150 mMNaCl; 1 mM ethylenediaminetetraacetic acid [EDTA]; 1 mM Na_2_EDTA; 1 mM ethylene glycol tetraacetic acid; 1% [v/v] Triton-X 100; 2.5 mM sodium pyrophosphate; 1 mMβ-glycerophosphate; 1 mM Na_3_VO_4_; and 1 μg/ml leupeptin and phenylmethylsulfonyl fluoride). Equal amounts of protein (20 μg) were separated by sodium dodecyl sulfate-polyacrylamide gel electrophoresis (SDS-PAGE; 12% separating gel). After electrophoresis, proteins were transferred to polyvinylidene difluoride membranes (Bio-Rad Laboratories, Tokyo, Japan). The blots were blocked with 1% casein in Tris-buffered saline (TBS) containing 0.1% Tween-20 (TBS-T) for 1 h at room temperature and then incubated with primary antibodies overnight at 4°C. Primary Abs against LMAN2 (described earlier) and β-actin (Sigma-Aldrich) were used. Membranes were washed in TBS-T and incubated with secondary horseradish peroxidase-labeled Ab for 1 h at room temperature. Bound Ab complexes were detected by enhanced chemiluminescence (Bio-RadLaboratories).

### Isolation of CD8^+^ cells from the spleens of GVHD-mediated rats

CD8^+^ cells from GVHD rats were used in transwell migration assays and Stamper-Woodruff binding assays. Lymphocytes were teased from spleen, exhibiting splenomegaly, of GVHD rats and suspended in RPMI-1640 medium at 4°C. The cells were dispersed by rapid, in-out pipetting, and the cell clumps were removed by passage through a nylon mesh (Cell Strainers; BD Biosciences). The resultant single-cell suspension was washed three times in the same medium and resuspended at a concentration of 3 × 10^7^mononuclear cells /ml. CD8^+^ cells from the suspension were isolated by magnetic bead purification using Miltenyi CD8a microbeads according to the manufacturer’s protocol (Miltenyi Biotec, Tokyo, Japan).

### Transwell migration assay for effector cells

Migration of CD8^+^ cells from the GVHD-spleen were evaluated in a transwell® insert system in which the top and bottom wells were separated by polycarbonate membrane (3-μm pore size; Corning, NY, USA). CD8^+^ cells, pre-incubated or not with anti-MBP Ab (50 μg/ml), were seeded at a density of 5 × 10^4^ cells /well onto 3-μm transwell inserts. The lower chamber was filled with 500 μl RPMI medium only or medium containing Man (2 mM), a mixture of Man and LCA (50 μg/ml), or galactose (Gal, 2 mM; EY Laboratories). The cells were incubated for 4 h at 37°C (5% CO_2_) and then stained with Diff-Quik (Sysmex Corp., Hyogo, Japan). Migration activity was evaluated as the percent migration of cells from the upper chamber of the transwell insert to the lower chamber in three high power fields (100x) per well. The experiment was performed in triplicate.

### Stamper-Woodruff binding assay (SWBA)

Stamper-Woodruff-type frozen-section assays were performed as described previously [3]. Briefly, aliquots containing 2.5 × 10^5^ isolated CD8^+^ cells in 200 μl of RPMI-1640 medium were added to freshly cut (8 μm) frozen sections of the tongue obtained from rats in the control and GVHD groups. The sections were agitated on a rotary shaker (60 rpm) for 60 min at room temperature. Next, the cell suspension was carefully decanted, and cells adhering to sections were fixed with 2.5% glutaraldehyde in PBS for 5 min. Slides were then washed in PBS and stained with 0.5% toluidine blue. The number of adherent CD8^+^ cells was determined by light microscopy examination of fields at 200 × magnification (Each high-power field represented approximately 400 μm of oral epithelium). The number of CD8^+^ cells attached directly over KCs (but not in the cornified layer) was counted. Several blocking experiments were performed as follow: (a) frozen sections of tongue from GVHD rats were incubated with 25 μg/ml of LCA, PNA or UEA-I for 30 min at room temperature following a brief wash with PBS, and then added to lymphocytes; (b) CD8^+^cells were preincubated with 50 μg/ml of MBP Ab for 30 min at room temperature, before the entire reaction mixture was transferred onto frozen sections of the GVHD-tongues; (c) the two approaches were used simultaneously, i.e., CD8^+^ cells were treated with MBP Ab and the tissue sections were treated with MBP. Results are calculated as the percentage of binding relative to control lymphocytes and tissue sections exposed to buffer alone.

### Statistical analysis

Statistical analysis was performed with one-way analysis of variance (ANOVA) and Scheffe’s multiple comparison tests to determine statistical differences among the samples. Data are presented as the mean ± standard deviation (SD) and *P* values of < 0.05 were considered statistically significant.

## Results

### Oral mucosal GVHD is characterized by ICAM-1 expression and CD8^+^ T-cell infiltrates

**Figure 1:**
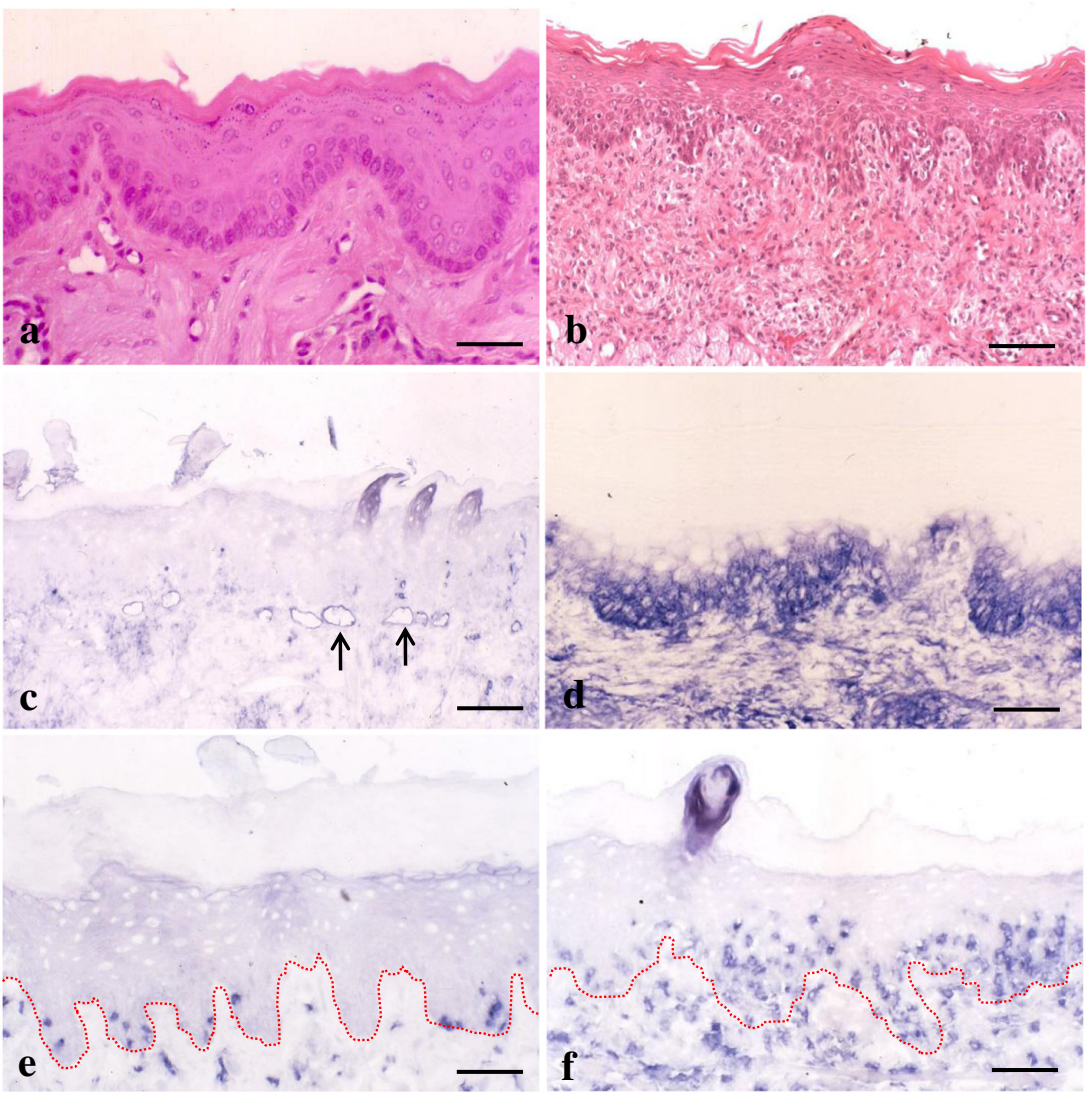
**Immunohistochemical expression of intercellular adhesion molecule-1 (ICAM-1) and CD8**^**+ **^**in graft-versus-host disease (GVHD)-related oral mucosa. a** and **b**: No obvious changes are observed in hematoxylin and eosin (H&E)-stained control tongue sections **(a)**. In tongue tissue sections from GVHD rats, epithelial destruction is observed **(b)**. **c** and **d**: In tongue specimens from control rats, ICAM-1 is expressed only in the vascular slits (arrows) **(c)**. Epithelial ICAM-1 expression is observed in the basal to spinous layers of the oral mucosa from GVHD rats **(d)**. **e** and **f**: Only a few CD8^+^ cells are found in the control **(e)**. In the GVHD rats, increased numbers of CD8^+^ cells are observed in both lamina propria and surface epithelium **(f)**. The dotted line shows a junction between the surface epithelium and lamina propria of the tongue. Bar = 100 μm.

First we examined whether immunohistochemical characteristics could be detected in the oral mucosa during the development of GVHD. In untreated control tongues, no histological changes were seen with H&E staining (Figure 1a). ICAM-1 expression was restricted to endothelial and perivascular cells of blood vessels (Figure 1c). Only a few CD8^+^ cells were observed in the lamina propria and oral epithelium (Figure 1e). The results were the same in tissue sections taken from experimental rats 8 days after injection. However, 10 days after induction of GVHD in LBNF_1_ rats, both histological and immunohistochemical changes were observed in the tongues. H&E staining revealed lesions typical of acute mucocutaneous GVHD, indicating eosinophilic cytoplasmic degeneration and necrosis of epithelial KCs (the so-called satellitosis) resulting from intraepithelial infiltration of lymphocytes (Figure 1b). ICAM-1 expression was observed in the basal to spinous layers of the epithelium (Figure 1d) and the number of CD8^+^ cells increased in the lamina propria of the tongue (Figure 1f). Furthermore, these cells were infiltrated into the surface epithelium where epithelial KCs were stained with anti-ICAM-1 Ab.

### Alterations in LCA binding in oral mucosal GVHD

**Figure 2:**
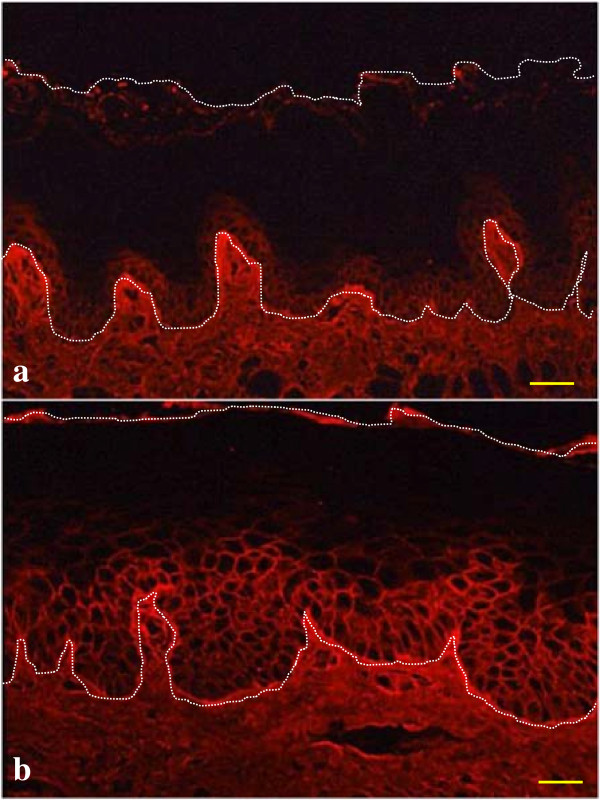
***Lens culinaris *lectin (LCA) binding in graft-versus-host disease (GVHD) oral mucosa.** LCA binding occurs on the cell surface of basal to parabasal cells in control tongues **(a)**. Staining extends to the spinous layers of the surface epithelium in GVHD rats **(b)**. The white dotted line circumscribes the surface epithelial layer of the tongue. Bar = 100 μm.

To elucidate whether histochemical expression of cell-surface Man on epithelial KCs is affected by the development of GVHD, we examined LCA staining in the tongues of control and GVHD rats. LCA binding was restricted to the cell surface of the basal to parabasal KCs in the surface epithelium of the tongues from control rats (Figure 2a). The same staining pattern was shown in tissue sections taken from experimental rats eight days after injection. In contrast, the level of LCA staining extended to the spinous layers of the surface epithelium 10 days after injection, indicating that oligosaccharides on KCs could be modified during the development of GVHD (Figure 2b). Furthermore, the extension pattern of LCA staining was identical to that of ICAM-1 expression.

### Increased expression of mannosyltransferase complex in oral mucosal GVHD

**Figure 3:**
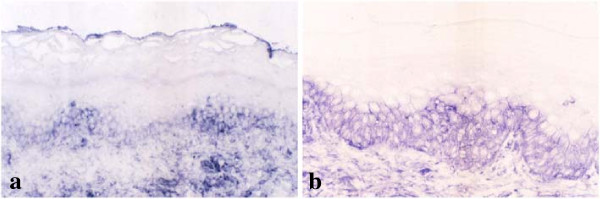
**Immunohistochemical detection of mannosyltransferase complex in graft-versus-host disease (GVHD)-affected oral mucosa.** ALG11, antibody (Ab) of mannosyltransferase complex, is weakly and faintly reactive in basal and parabasal epithelial cells of control tongues **(a)**. In contrast, staining with ALG11 extends to keratinocytes (KCs) in the spinous layers of tongues from GVHD rats **(b)**. Bar = 100 μm.

We next examined the immunohistochemical expression of mannosyltransferase complex, using ALG11 Ab, in tongues from both control and GVHD rats, to elucidate whether the mannosyltransferase could be responsible for the addition of Man on the oligosaccharides in KCs during the development of GVHD. In normal tongue, ALG11 bound weakly and/or faintly to basal and parabasal KCs of the surface epithelium (Figure 3a). Reactive sites with ALG11 extended to the cytoplasm of KCs in the spinous layers of the surface epithelium in the tongues of GVHD rats (Figure 3b). The reactive range with ALG11 seemed to be identical to that of LCA binding, suggesting that increased expression of mannosyltransferase in the epithelial layers may be related to extensive binding of LCA.

### MBP /Man-binding pathway induces CD8^+^ cell migration in vitro

**Figure 4:**
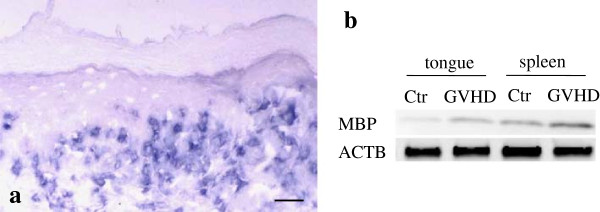
**Mannose-binding protein (MBP) expression in tongue and spleen specimens of graft-versus-host disease (GVHD) rats.** Infiltrating cells are expressed immunohistochemically by anti-MBP antibody (Ab) **(a)**. Western blot analysis of MBP levels of the tongue and spleen from control and GVHD rats **(b)**. Both tongue and spleen from the GVHD-mediated rats show remarkable reaction with MBP. β-actin (ACTB) was similarly analyzed as a loading control. Bar = 100 μm.

**Figure 5:**
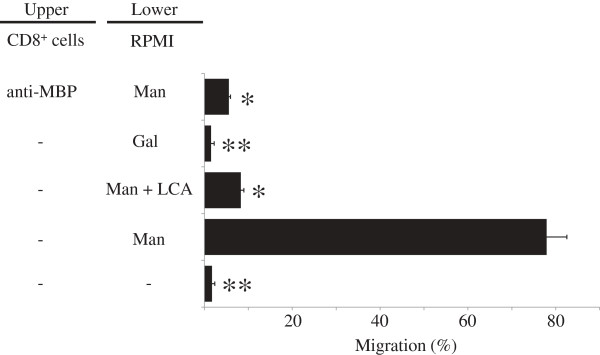
**Induction of mannose (Man) to CD8**^**+ **^**cell migration.** CD8^+^ cell migration was examined by the transwell migration assay. Man, Man with *Lens culinaris* lectin (LCA), or galactose was placed in the lower chamber well. The wells of the upper chamber receive isolated CD8^+^ cells, pretreated or not with anti-mannose-binding protein (MBP) antibody (Ab). RPMI was used as a negative control. The figure represents results of three different experiments expressed as mean ± standard deviation. Same symbols show no statistically significant differences. Others, significantly different at *P* < 0.05.

To elucidate if migration of CD8^+^ cells is mediated by the MBP/Man-binding pathway, we examined MBP expression in the tongues of GVHD rats and the transwell migration assay for CD8^+^ cells. First, we examined immunohistochemical detection of MBP in tissue sections of the tongue from the GVHD-rats. MBP^+^ infiltrating cells were observed in both the upper lamina propria and surface epithelium of the tongues (Figure 4a). We also examined protein expression of MBP in the tongues and spleen of control and GVHD rats by Western blot analysis. As shown in Figure 4b, the amount of MBP accumulation was markedly increased in both the tongue and spleen of GVHD rats. In contrast, expression of MBP protein was faint or negative in both the tongue and spleen of control rats (Figure 4b). These results suggest that MBP may be induced on GVHD-related effector cells and MBP on effector cells may react against cell-surface Man of KCs in the tongues of GVHD rats. To test this hypothesis, we next performed transwell migration assays for effector cells. As shown in Figure 5, the percent migration of CD8^+^ cells to Man increased drastically (77.9 ± 4.6) compared with control (1.7 ± 0.6). The migration percent of CD8^+^ cells became lower in the lower chamber containing Man and LCA (8.3 ± 0.7) and Gal (1.5 ±0.7). Furthermore, the CD8^+^ cell migration to Man decreased drastically (5.6 ± 0.4) when the cells were pre-incubated with anti-MBP Ab. These results indicate that migration of CD8^+^ cells to Man is mediated by the MBP/Man-binding pathway.

### Anti-MBP Ab and LCA inhibit adhesion of CD8^+^ cells to KCs in tongue specimens from rats with GVHD

**Figure 6:**
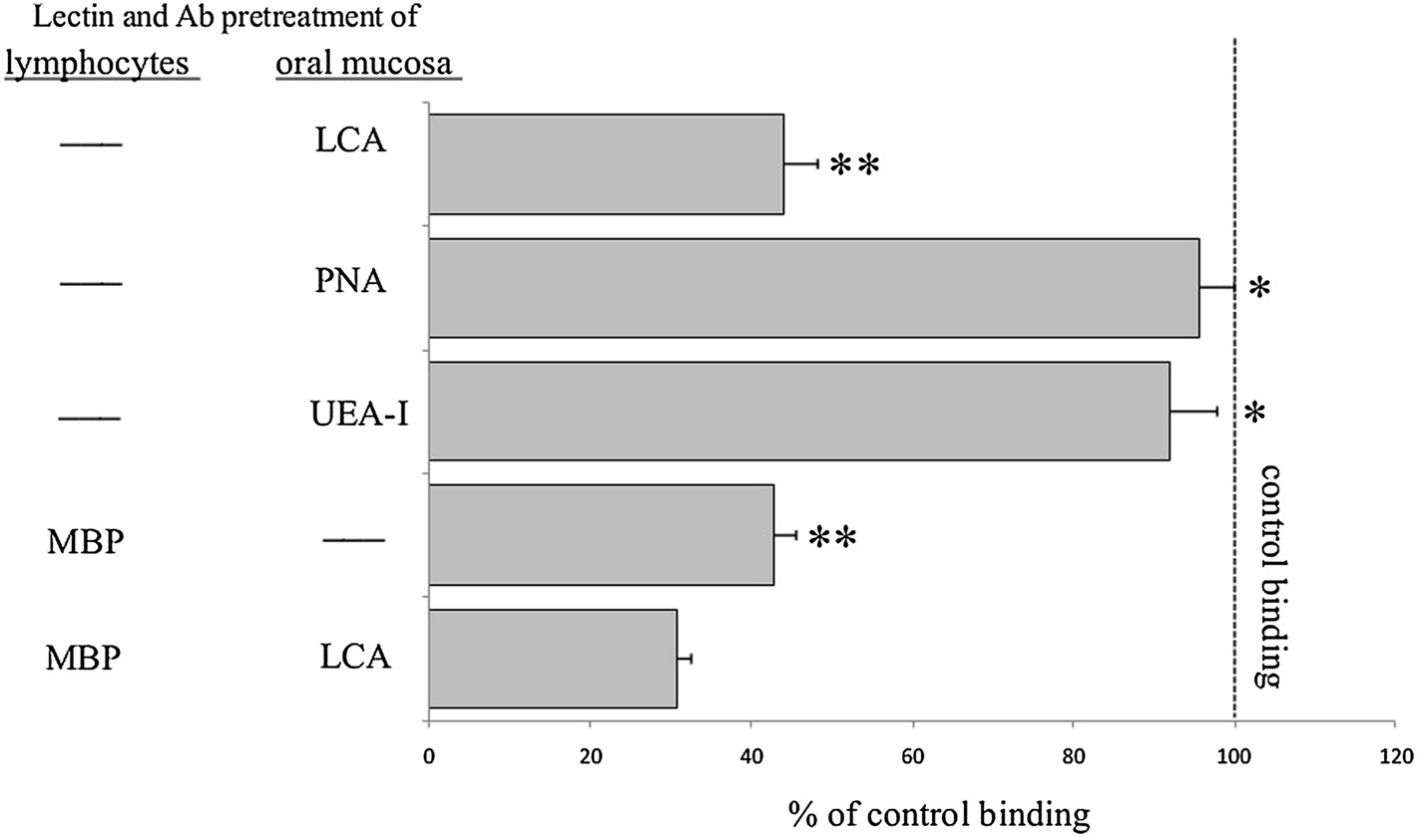
**Effect of lectins and anti-mannose-binding protein (MBP) antibody (Ab) on CD8**^**+ **^**cell adhesion to the oral epithelium in GVHD.** Adhesion of CD8^+^ cells to the oral keratinocytes (KCs) were investigated by Stamper-Woodruff binding assay (SWBA). Binding of CD8^+^ cells was significantly decreased when the cells were pretreated with anti-MBP Ab, as well as when epithelia were pretreated with *Lens culinaris* lectin (LCA). The figure represents the results of three different experiments and expressed as mean ± standard deviation. Same symbols show no statistically significant differences. Others, significantly different at *P* < 0.05.

In the rat GVHD model, effector cells can bind to lesional KCs by the ICAM-1/LFA-1 pathway [3]. We performed SWBA using CD8^+^ cells and/or oral epithelial cells taken from rats with GVHD, to provide evidence of a direct role of the MBP/Man-binding pathway in the binding of CD8^+^ cells to epithelial KCs. Pretreatment of the tissue sections prepared from the oral mucosa with PNA and UEA-I had no effect, whereas LCA decreased lymphocyte adhesion by 42.3% of the control value (Figure 6). When lymphocytes from rats with GVHD were incubated with anti-MBP Ab, lymphocyte adhesion to epithelial KCs was 42.7% of the control value (Figure 6). When the two approaches were applied simultaneously, i.e., when the lymphocytes were treated with anti-MBP Ab and the tissue sections were treated with LCA, lymphocyte adhesion was decreased to <40% of the control value (Figure 6). These results indicate that the adhesion of CD8^+^ cells to epithelial KCs is mediated in part by the MBP/Man-binding pathway.

## Discussion

In this study we use the haploidentical allogeneic F_1_ hybrid rat model to elucidate the role of cell-surface expression of Man residues by KCs in GVHD. Although this model does not directly represent clinical hematopoietic cell transplantation in human, it provides a convenient and genetically well-defined system from which to gain insights into the mechanisms underlying immune-mediated tissue damages of host epithelial tissue by studying the *in vivo* interactions between KCs and lymphocytes [17]. We present three lines of evidence to support the conclusion that cell-surface expression of Man by KCs plays a role in the migration and/or adhesion of CD8^+^ cells in the epithelium during the development of GVHD. First, a lectin histochemical approach confirmed that the cell-surface expression of Man by KCs was up-regulated in the development of GVHD. Second, protein expression and transwell migration results indicated that CD8^+^ cell migration to Man was related to an interaction between MBP and Man. Third, CD8^+^ cell adhesion of the mucosal surface epithelium was mediated by the MBP/Man-binding pathway as revealed using SWBA.

An up-regulation of LCA binding on the cell surface of KCs indicates that the cell-surface expression of Man is modified during the development of oral mucosal GVHD. In mucocutaneous GVHD, specific molecular changes occur in KCs to allow lymphocyte migration into the surface epithelial layer [7,8,18]. A previous study has reported that the induction of ICAM-1 expression on KCs leads to the migration of CD8^+^ cells into the epithelium and that this process is mediated in part by the ICAM-1/LFA-1 pathway [3]. The results presented here provide additional support for molecular changes in KCs during the development of oral mucsal GVHD. The immunohistochemical study of anti-ALG11 Ab reported here shows that Ab reactive sites also expand to spinous layers in the surface epithelium in the GVHD tongue. ALG11 is known as alpha-1,2-mannosyltransferase in N-linked glycoprotein and is localized to the cytosolic side of the endoplasmic reticulum in basal cells of normal squamous epithelia [19]. These findings suggest that the extensive expression of mannosyltransferase complex may be indirectly responsible for the upregulation of Man expression on the cell surface of KCs in the GVHD tongue. Future work will address why the expression of mannosyltransferase complex and Man expand during the development of GVHD.

Immunohistochemical and Western blot analyses presented here reveal that effector cells of GVHD show the expression of MBP using LMAN2 Ab, in addition to CD8. MBP, also called mannan-binding protein or mannan-binding lectin, is a Ca2^+^-dependent (C-type) animal lectin [20-22]. A recent study revealed that this protein could display a specific effect on T cell activation [23]. MBP preferentially binds to carbohydrates terminated with Man, N-acetylglucosamine, or fucose [24]. Therefore, MBP-expressing lymphocytes seem to interact with Man-expressing KCs in oral mucosal GVHD. Our results of the transwell migration assay indicate that the migration of the MBP-expressing CD8^+^ cells is accelerated by medium containing Man. CD8^+^ cell migration was inhibited by a mixture of Man and LCA and pretreatment of cells with LMAN2 Ab, indicating that MBP on CD8^+^ cells recognized Man specifically. These results suggest that the migration of effector cells, CD8^+^ lymphocytes, to Man-expressing KCs may be mediated by an interaction between MBP and Man.

CD8^+^ cell-adhesion to epithelium stained with Man-specific LCA was demonstrated using SWBA. Thus, the binding of CD8^+^ cells on the surface epithelium during the development of GVHD increases along with Man expression and correlates with disease progression. These results are consistent with *in vivo* observations of KCs expressing Man-specific LCA to adhere to CD8^+^ cells during the progression of GVHD. In an adhesion process, MBP on CD8^+^ cells plays an important role as an adhesive factor of Man-expressing KCs during the development of GVHD. Furthermore, the results of LCA and Ab-blocking experiments supported the idea that MBP/Man interactions account for the binding of CD8^+^ cells. Thus, LMAN2 Ab, LCA, or LMAN2 Ab together with LCA, blocked all CD8^+^ cell-binding compared with lecitns recognizing unrelated Man residues. Our previous study revealed that the induction of ICAM-1 expression on KCs led to the adhesion of CD8^+^ cells to the surface epithelium and that this was mediated in part by the ICAM-1/LFA-1 pathway [3]. The MBP/Man interactions presented here belong to another adhesive pathway for the migration and adhesion of effector cells in the surface epithelium during the development of oral mucosal GVHD.

In summary, these results define the role of Man expression on KCs during the development of GVHD as inducing migration of MBP-expressing CD8^+^ cells into the oral epithelium where they adhere to KCs. The upregulation of Man expression on KCs is undoubtedly a key step in the pathogenesis of oral mucosal GVHD.

## Conclusion

An increased expression of Man on KCs leads to the migration and/or adhesion of CD8^+^ cells in the surface epithelium and this is mediated in part by the MBP/Man pathway during the development of oral mucosal GVHD.

## Abbreviations

ACTB: β-actin; Ab: Antibody; GVHD: Graft-versus-host disease; H&E: Hematoxylin and eosin; ICAM-1: Intercellular adhesion molecule-1; KCs: Keratinocyte; LCA: *Lens culinaris* lectin; LFA-1: Lymphocyte function-associated antigen-1; MBP: Mannose-binding protein; Man: Mannose; PBS: Phosphate-buffered saline; SWBA: Stamper-Woodruff binding assay.

## Competing interests

The authors declare that they have no competing interests.

## Authors’ contributions

HH and JO planned the study, performed the experiments and data analysis, and wrote the manuscript. KS and NO performed the lectin staining and helped to draft the manuscript. KT supervised writing of the manuscript. All authors read and approved the final manuscript.

## Pre-publication history

The pre-publication history for this paper can be accessed here:

http://www.biomedcentral.com/1472-6831/14/5/prepub
